# GE11 Peptide Conjugated Liposomes for EGFR-Targeted and Chemophotothermal Combined Anticancer Therapy

**DOI:** 10.1155/2021/5534870

**Published:** 2021-03-31

**Authors:** Xueqin Huang, Lingzhi Chen, Yuping Zhang, Suyan Zhou, Huai-Hong Cai, Ting Li, Hua Jin, Jiye Cai, Haibo Zhou, Jiang Pi

**Affiliations:** ^1^State Key Laboratory of Quality Research in Chinese Medicines, Macau University of Science and Technology, Macau 000583, China; ^2^Department of Chemistry, Jinan University, Guangzhou 510632, China; ^3^Department of Clinical Immunology, Institute of Clinical Laboratory Medicine, Guangdong Provincial Key Laboratory of Medical Molecular Diagnostics, Guangdong Medical University, Dongguan, Guangdong 523808, China; ^4^Institute of Pharmaceutical Analysis, College of Pharmacy, Jinan University, Guangzhou, Guangdong 510632, China

## Abstract

How to actively target tumor sites manipulating the controllable release of the encapsulated anticancer drugs and photosensitizers for synergistic anticancer therapy remains a big challenge. In this study, a cancer cell-targeted, near-infrared (NIR) light-triggered and anticancer drug loaded liposome system (LPs) was developed for synergistic cancer therapy. Photosensitizer indocyanine green (ICG) and chemotherapy drug Curcumin (CUR) were coencapsulated into the liposomes, followed by the surface conjugation of GE11 peptide for epidermal growth factor receptor (EGFR) targeting on the cancer cell surface. Strictly controlled by NIR light, GE11 peptide modified and CUR/ICG-loaded LPs (GE11-CUR/ICG-LPs) could introduce hyperthermia in EGFR overexpressed A549 cancer cells for photothermal therapy, which could also trigger the increased release of CUR for enhanced cancer cell inhibition. GE11-CUR/ICG-LPs synergized photochemotherapy could induce reactive oxygen species (ROS) generation and cytoskeleton disruption to activate stronger apoptotic signaling events than the photothermal therapy or chemotherapy alone by regulating Bax/Bcl-2 and PI3K/AKT pathways. This EGFR-targeted drug-delivery nanosystem with NIR sensitivity may potentially serve in more effective anticancer therapeutics with reduced off-target effects.

## 1. Introduction

As one of the leading causes of death, cancer is accounting for millions of deaths annually worldwide [1]. Despite the rapid advances in recent therapy techniques, chemotherapy is still the principal cancer treatment approach in the clinic. Curcumin (CUR) has been widely investigated for its anticancer properties and has proven excellent anticancer effects in several clinical trials [2]. Unfortunately, the low cancer targeting effect, low bioavailability, poor aqueous solubility, and fast metabolism still restrict the anticancer effects of CUR, which further hinders its clinical use. Thus, how to enhance the anticancer effects of CUR by some novel strategies remains the most urgent challenge for its further clinical application.

Drug-delivery systems based on nanomaterials, such as microemulsion [3, 4], micelles [5, 6], nanogels [7, 8], and dendrimers [9, 10], have been designed and developed to potentially overcome the low anticancer effects of drugs. Owing to the fascinating physical and chemical properties, liposomes (LPs) have been extensively used to improve the solubility and bioavailability of CUR, attracting substantial attention in the past decades [11]. Poly(ethylene glycol) polymer (PEG) modification enables liposomes to effectively escape from immune recognition and clearance in blood, thus greatly improving their stability and prolonging the blood circulation time [12]. However, PEGylated liposomes are still difficult to specifically accumulate at the tumor site due to the lack of active tumor-targeting effects. As a result, countless research efforts have been devoted to constructing functional LPs with specific surface modifications, such as folic acid [13], antibodies [14], hyaluronic acid [15], and cRGD [16] for active cancer cell targeting. Our and other groups' work have demonstrated that the synthetic 12 amino acids peptide GE11, with the sequence of Y-H-W-Y-G-Y-T-P-Q-N-V-I, was an efficient epidermal growth factor receptor (EGFR) targeting peptide for EGFR overexpressed cancer cells [17–19]. After modifying with GE11 peptide, the obtained LPs are expected to selectively deliver loading drugs into cancer cells for enhanced anticancer treatment. However, typical nanocarriers always release drugs passively with incomplete and poorly controlled release in the target sites [20], which urges us to develop nanosystems with on-demand release properties at the desired sites for more effective anticancer treatment.

Over the past few years, light-responsive [21–23] and other stimuli responsive [24–26] drug-delivery nanocarriers are widely developed to trigger drug release, which undoubtedly provides a feasible strategy to maximize the drug concentration at tumor sites and minimize off-target drug exposure. Near-infrared (NIR) laser-sensitive nanocarriers, which exhibit strong absorption in the NIR region (700–900 nm), have received widespread attention [21]. Heat converted from the absorbed NIR light energy can not only destroy the nanocarriers for drug burst release but ablates cancer cells robustly, which is thus highlighted as a promising strategy for cancer treatment. Although gold or inorganic nanoparticle-based light-triggered nanosystems have been widely reported [27, 28], their potential toxicity in various tissues is still a big issue for further clinical uses. In contrast, indocyanine green (ICG), a NIR photosensitizer for clinical use approved by the US Food and Drug Administration (FDA), is proposed as a novel phototherapy agent owing to its biocompatibility and remarkable NIR optical features [29]. In recent years, nanocarriers are expected to improve the disadvantages of ICG molecules, such as the short blood half-time and the instability for more effective phototherapy, which also enables more effective cancer inhibition by NIR light irradiation.

In this study, we fabricated a NIR light-triggered drug release nanosystem for the combination of photo- and chemotherapy based on our nanotechnology expertise [19, 30–33]. Photosensitizer ICG and chemotherapy drug CUR were coencapsulated into the liposomes, whose surface was further modified with GE11 peptides (GE11-CUR/ICG-LPs), to endow it with active targeting effects toward EGFR-positive-expressed cancer cells. Upon 808 nm NIR laser irradiation, GE11-CUR/ICG-LPs could be destroyed by hyperthermia produced by ICG to release encapsulated CUR after GE11 peptide targeted delivery to the tumor site. This novel nanosystem could ablate the tumor by hyperthermia, and the NIR triggered release of CUR would further eradicate residual cancer cells, thus achieving synergetic anticancer effects. This work presents a novel strategy to combine photo- and chemotherapy by controllable nanodelivery system, which shows strong potentials for more effective anticancer treatment.

## 2. Materials and Methods

### 2.1. Materials

3-(4,5-dimethylthiazol-2-yl)-2,5-diphenyltetrazolium bromide (MTT) was obtained from Sigma Aldrich (USA). Dulbecco's modified eagle medium (DMEM), fetal bovine serum (FBS), trypsin-EDTA (0.05%), penicillin-streptomycin (P/S), PBS, calcein-AM, and enhanced chemiluminescence (ECL) western blotting detection reagent were purchased from Thermo Fisher Scientific (USA). GE11 polypeptide was purchased from GL Biochem Ltd. (Shanghai, China). Radioimmunoprecipitation assay (RIPA) lysis buffer, the primary antibodies of anti-PI3K (4292s), anti-p-PI3K (4228s), anti-Akt (9272s), anti-p-Akt (4060s), anti-Bcl-2 (3498s), and anti-Bax (5023s), were purchased from Cell Signaling Technology (USA). Bio-Rad protein assay kit and 30% acrylamide/bissolutionandpolyvinylidene difluoride (PVDF) membrane were obtained from Bio-Rad (USA). Annexin V-FITC/PI apoptosis detection kit, reactive oxygen species (ROS) detection kit, paraformaldehyde, 4,6-diamidino-2-phenylindole (DAPI), and Tubulin-Tracker were purchased from Beyotime Institute of Biotechnology (Shanghai, China).

### 2.2. Preparation and Characterization of GE11-CUR/ICG-LPs

Liposomes loaded with CUR/ICG (CUR/ICG-LPs) were prepared using the film dispersion method followed by membrane extrusion. Briefly, the hybrid phospholipid (SPC, cholesterol, DSPE-PEG2000, and DSPE-PEG2000-MAL (60 : 20 : 10 : 10, w/w) and 0.5 mg CUR were dissolved in a mixed solution (methanol: chloroform = 1, v/v) and evaporated to form a thin lipid film in a round-bottom flask using a rotary evaporator. Then, the film was vacuum-dried inside the desiccator overnight to remove residual solutions. The film was hydrated with a PBS solution containing ICG (0.4 mg) by sonication in a water bath for more than 10 min. Subsequently, the mixture was subjected to emulsification using an ultrasonicator at a power of 50 W for 10 min (5 s start, 5 s stop), followed by extrusion through a series of polycarbonate membranes with the pore size ranging from 400 nm to 100 nm by an Avanti miniextruder (Millipore). After that, free ICG and CUR were separated from liposomes by centrifugation at 20000 g for 30 min with an ultrafiltration tube (30 kD, Millipore).

To obtain GE11-CUR/ICG-LPs, CUR/ICG-LPs were mixed with 1-ethyl-3- [3-dimethylaminopropyl] carbodiimide hydrochloride (EDC, 0.4 M) and N-hydroxysuccinimide (NHS, 0.1 M) then kept for 3 hours at 4°C. GE11 peptides (18.6 mg) were then added into the mixture and incubated overnight at room temperature in the dark. The unconjugated GE11 was removed using ultrafiltration tubes against distilled water. To obtain CUR-loaded LPs (GE11-CUR-LPs) or ICG-loaded LPs (GE11-ICG-LPs), all preparation processes were similar except that ICG solution or CUR solution was not added into the mixture.

### 2.3. Characterization and Stability of GE11-CUR/ICG-LPs

The encapsulation efficiency (EE) of CUR and ICG in GE11-LPs was detected by UV/vis spectrometry at 416 and 790 nm according to the standard curve, respectively. EE (%) = ((Mt ‒ Mul)/Mt) × 100% (Mt: the total amount of CUR or ICG in the formulation; Mul: the amount of the unloaded CUR or ICG). The particles size, size distribution, *ζ*-potential, and morphology were further determined using a Malvern Zetasizer Nano ZS90 instrument (Malvern Instruments Ltd., Malvern, UK) and transmission electron microscope (TEM), respectively. The optical properties were measured using an ultraviolet visible (UV-Vis) spectroscopy (PerkinElmer, Waltham, MA) and Fourier transform infrared spectroscopy (FTIR, Bruker TENSOR27, Germany). The absorption and fluorescence stability were assessed at predetermined times using UV/vis and fluorescence spectrometry.

The photothermal properties were further investigated by monitoring the temperature change. Briefly, quartz cuvettes containing free CUR/ICG, CUR/ICG-LPs, and GE11-CUR/ICG-LPs were treated with 1 Wcm^−2^ 808 nm laser irradiation for 5 min. The laser was adjusted to ensure the irradiation point can cover the whole surface of the samples, and the temperature was recorded using an infrared camera (FLIR ONE Pro). For further comparing the photostability between free CUR/ICG and GE11-CUR/ICG-LPs, the samples were irradiated for four cycles of laser on/off with 808 nm laser irradiation. Additionally, the size distribution of the irradiated samples was examined after the last NIR irradiation.

### 2.4. Drug Release Property of GE11-CUR/ICG-LPs

The CUR release behavior from GE11-CUR/ICG-LPs with or without NIR irradiation (808 nm) at 1 Wcm^−2^ for 5 min was determined by dialysis method in PBS. Briefly, GE11-CUR/ICG-LPs were loaded into a dialysis bag filter (MWCO: 8000 Da) tightly sealed, and then immersed into 30 mL release media with mild shaking at 50 rpm. For NIR irradiation, GE11-CUR/ICG-LPs were taken out of the dialysis bag and irradiated by NIR laser at 808 nm. After irradiation, the samples were put back into the dialysis bag to continue the release study. At predetermined time intervals, 0.3 mL release medium was sampled and replaced with an equal volume of fresh release medium. Then the concentrations of the CUR released profiles were determined on the absorbance intensity at 416 nm, using a standard calibration curve obtained.

### 2.5. Cell Culture

A549 nonsmall cell lung cancer cells line, HeLa human cervical cancer cell and LO2 human normal liver cells were purchased from American Type Culture Collection (ATCC, USA). The cells were cultured in DMEM medium containing 10% FBS, 100 U·mL^−1^ penicillin, and 100 *μ*g·mL^−1^ streptomycin in a humidified incubator at 37°C and adjusted to 5% CO_2_.

### 2.6. Intracellular Uptake and Light-Simulated Drug Release of GE11-CUR/ICG-LPs

A549 and LO2 cells were seeded (3 × 10^5^ cells/well) and incubated on coverslips in a 6-well plate for 24 h and then subjected to a fresh medium containing free CUR/ICG, CUR/ICG-LPs, and GE11-CUR/ICG-LPs (CUR: 12.5 *μ*g·mL^−1^, ICG: 10 *μ*g·mL^−1^). After incubation for 4 h, the cells were washed with PBS for 3 times, fixed with 4% paraformaldehyde, and stained with DAPI (5 *μ*g/ml) and finally observed on Leica SP8 confocal microscope equipped with a 40 × oil immersion objective (Leica, Germany).

In order to detect light-triggered release behavior of the GE11-CUR/ICG-LPs, A549 cells were pretreated with GE11-CUR/ICG-LPs (CUR: 12.5 *μ*g·mL^−1^, ICG: 10 *μ*g·mL^−1^) for 4 h, followed by irradiation with 808 nm NIR laser (1 Wcm^−2^) for 0, 5, 10 min. After that, the cells were washed, fixed, stained, and then observed on Leica SP8 confocal microscope (Leica, Germany).

### 2.7. Western Blot Analysis

The protein corona was characterized using the sodium dodecyl sulfate-polyacrylamide gel electrophoresis (SDS-PAGE) method. GE11-LPs were incubated at culture medium for 12 h and centrifuged to collect GE11-LPs that contained protein corona, then repeatedly washed in cold PBS to remove unbound proteins. The Bradford protein assay was carried out on the washing solution to ensure that no additional proteins were eluted from GE11-LPs. The GE11-LPs not incubated at culture medium were subjected to the same procedure as a control to verify the absence of protein corona. All the samples were mixed with loading buffer (5X) and heated to 100°C for 10 min. Afterward, identical volumes (20 *μ*l) of each sample were loaded on the well with 10% SDS-PAGE gel and were run at 100 V for about 150 minutes. The proteins were then stained with Commassie Blue for 2 h and imaged after washing for 24 h. For western blot analysis, the treated cells were harvested and lysed at 4°C with a RIPA lysis buffer containing a mixture of protease and phosphatase inhibitor for 15 min. After centrifugation (12000 rpm, 15 min) at 4°C, the supernatant was collected as the cell lysates for protein content analysis by Bradford protein assay. Equivalent protein (30 *μ*g) from each sample was subjected to SDS-PAGE and then transferred to PVDF membranes (0.2 *μ*m). After blocking with 5% BSA, PVDF membrane was incubated with the following primary antibodies (1 : 1000): EGFR, PI3K, p-PI3K, Akt, p-Akt, *β*-actin, Bcl-2, and Bax at 4°C overnight. After extensively washed, the membrane was further incubated with secondary antibody (1 : 5000) for 4 h and then visualized by ECL reagents.

### 2.8. ROS Generation Assay

The intracellular reactive oxygen species (ROS) was detected using a ROS probe (DCFH-DA). Briefly, A549 cells grown on coverslips were grown in a fresh medium containing free CUR/ICG, CUR/ICG-LPs, and GE11-CUR/ICG-LPs (CUR: 12.5 *μ*g·mL^−1^, ICG: 10 *μ*g·mL^−1^) for 12 h, followed by treatment with 808 nm irradiation (1 W·cm^−2^, 5 min). After that, cells were washed twice with PBS, cultured with a serum-free medium containing 40 *µ*M DCFH-DA for 30 min. Finally, the cells were observed on Leica SP8 confocal microscope (Leica, Germany).

### 2.9. Immunofluorescence Assay of EGFR

HeLa, A549, and LO2 cells were washed with PBS and then fixed in 4% PFA for 15 min, followed by permeabilization with 0.1% Triton X-100 in PBS for 10 min. The samples were blocked with 3% BSA for 1 h, incubated overnight at 4°C with the EGFR antibodies (1 : 100), and then washed and incubated with secondary antibody (Alexa Fluor 488 goat anti-rabbit, Invitrogen, 1 : 200) for 2 h. Finally, the cells were observed on Leica SP8 confocal microscope (Leica, Germany).

### 2.10. Intracellular Microtubulin Distribution Analysis

A549 cells grown on coverslips were treated with free CUR/ICG, CUR/ICG-LPs, and GE11-CUR/ICG-LPs (CUR: 12.5 *μ*g·mL^−1^, ICG: 10 *μ*g·mL^−1^) for 12 h, followed by treatment with 808 nm irradiation (1 W·cm^−2^, 5 min). Afterward, the cells were incubated with Tubulin-Tracker Red (1 : 100) for 1 hour at room temperature and then washed with PBS. The entire nucleus was stained with DAPI (5 *μ*g/ml) for 15 min. After extensive rinsing with PBS, the cells were observed on Leica SP8 confocal microscope (Leica, Germany).

### 2.11. Cell Viability Analysis

A549 cells (8 × 10^3^ cells/well) were seeded into a 96-well plate and then subjected to a fresh medium containing free CUR/ICG, CUR/ICG-LPs, and GE11-CUR/ICG-LPs (CUR: 15 *μ*g·mL^−1^, ICG: 12 *μ*g·mL^−1^) for 12 h, followed by treatment with or without 808 nm irradiation (1 W·cm^−2^, 5 min). The laser spot area is ≈1 cm^−2^, which is enough to cover the bottom of the well on the 96-well plate. After further incubation for 12 h, the MTT assay was performed according to the reported method [34]. For the MTT assay, the medium was replaced by a fresh medium (100 *μ*L) containing MTT (0.5 mg/mL) and incubated for another 3 h. After that, the medium was removed and replaced by 150 *μ*L dimethyl sulfoxide (DMSO). The absorbance of formazan for each well at 520 nm was measured by a SpectraMax Paradigm multimode microplate reader (Molecular Devices, Sunnyvale, CA, USA) to determine cell viability. The relative cell viability (%) for each sample was presented relative to the control cells.

The effects of chemophotothermal combination therapy with different concentrations were also evaluated. A549 cells were subjected to a fresh medium containing free CUR/ICG, CUR/ICG-LPs, and GE11-CUR/ICG-LPs with different concentrations (CUR: 5, 10, 15, 20,25 *μ*g·mL^−1^ corresponds to ICG: 4, 8, 12, 16, 20 *μ*g·mL^−1^) for 12 h, followed by treatment with or without 808 nm irradiation (1 W·cm^−2^, 5 min). After further incubation for 12 h, the cell viability was determined by a MTT assay as described above.

For calcein-AM staining, the medium was replaced with a fresh medium containing calcein-AM. After incubation for 30 min at room temperature in the dark, cells were rinsed again with a fresh culture medium, and the live cells were observed on an Olympus IX71 microscope.

### 2.12. Caspase-3 Activity Analysis

The activity of caspase-3 was determined by fluorescence quantification according to manufacturer's instructions (Beyotime Institute of Biotechnology, China). Briefly, cells were incubated with free CUR/ICG, CUR/ICG-LPs, and GE11-CUR/ICG-LPs for 12 h, followed by treatment with 808 nm irradiation (1 W·cm^−2^, 5 min), then harvested by scraping, lysed in the lysis buffer, and finally centrifuged to remove cell debris. The supernatant was immediately measured for protein concentration and caspase activity. The cell lysates were then incubated with a specific caspase-3 substrate (Ac-DEVD-pNA) in reaction buffer for 2 h at 37°C. Absorbance at 405 nm was measured using a microplate reader (Molecular Devices, USA).

### 2.13. Statistical Analysis

All experiments were carried out at least in triplicate and all data were presented as mean ± SD. Statistics were performed using ANOVA analysis (Applied when more than two groups were analyzed) and t-test (Applied when just two groups were analyzed) with *P* < 0.05 regarded as statistically significant.

## 3. Results and Discussion

### 3.1. Preparation and Characterization of GE11-CUR/ICG-LPs

**Figure 1:**
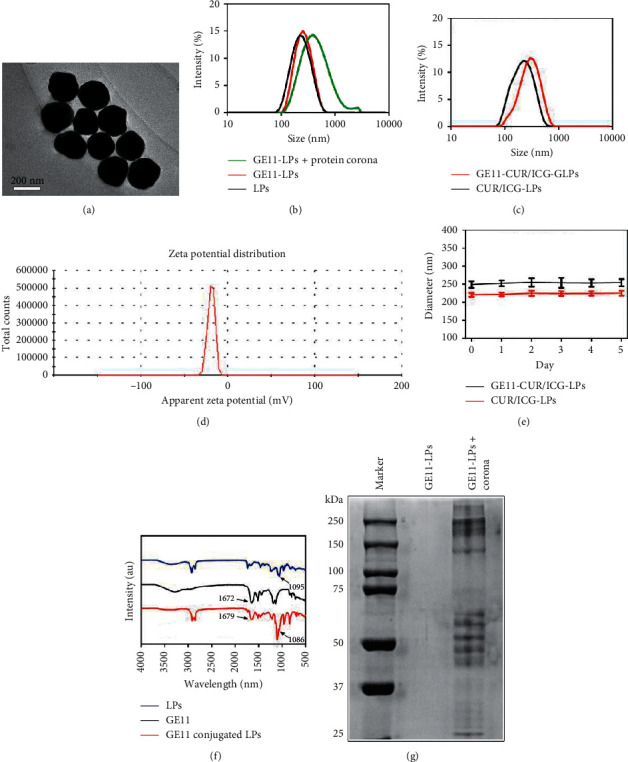
(a) TEM images of GE11-CUR/ICG-LPs. Scale bar = 200 µm. (b) Size distribution of LPs, GE11-LPs, and GE11-LPs + protein corona. (c) Size distribution of CUR/ICG-LPs and GE11-CUR/ICG-LPs. (d) Zeta potential of GE11-CUR/ICG-LPs. (e) Time-dependent diameter changes of CUR/ICG-LPs and GE11-CUR/ICG-LPs in PBS at 37°C. (f) FTIR spectra of LPs, GE11 peptide, and GE11-LPs. (g) Identification of protein corona absorbed on the surface of GE11-LPs by SDS-PAGE.

Here, photosensitizer ICG and chemotherapeutic agent CUR were coencapsulated into liposomes through film hydration, followed by conjugation with GE11 peptides for EGFR-positive A549 cancer cell targeting. Dynamic light scattering (DLS) measurements showed that LPs were ∼220 nm spherical particles with homogeneous distribution (Figure 1(b)), which was suitable for passive targeting of the tumor through the EPR effect [35]. Conjugations of GE11 peptides slightly increased LPs sizes to ∼250 nm, indicating successful decoration of targeting ligands on the surfaces of the LPs which increased the particle size of the LPs. However, there was no significant change in the particle size when ICG and CUR were coencapsulated into the GE11-LPs (Figures 1(a) and 1(c)). Both nanoparticles were dispersed uniformly with polydispersity index (PDI) values less than 0.3. And the sizes of CUR/ICG-LPs and GE11-CUR/ICG-LPs in PBS were almost unchanged within 5 days of storage (Figure 1(e)), indicating the high stability of the obtained nanosystem.

The zeta potential of all nanoparticle formulations was negative, while GE11-CUR/ICG-LPs was the most negatively charged system with a zeta potential of −19.0 mV (Figure 1(d)). In addition, hydrophobic CUR could be encapsulated into the hydrophobic layer of liposomes with high encapsulation efficiency (85.3%), which dramatically improved the solubility and stability of CUR. The drug-loading capacity of the GE11-CUR/ICG-LPs formulation was 5.70 % and 7.12 % for ICG and CUR, respectively.

The liposomes containing DSPE-PEG2000-MAL could covalently bind to the COOH groups of GE11 peptide to form GE11-LPs. The chemical structure of GE11 conjugated LPs was further verified by FTIR. As shown in Figure 1(f), the characteristic peak of GE11-LPs at 1086 cm^−1^ was due to the stretching vibration of C-O-C, which was similar to the specific absorption of the PEG segment at 1095 cm^−1^. And, the absorbance peak at 1679 cm^−1^ could be attributed to the stretching vibration of C(=O)-N, which was similar to the specific absorption of GE11 peptide at 1672 cm^−1^, confirming the successful conjugation of GE11 peptide onto LPs. By modification of GE11 peptide, the as-prepared LPs have improved the therapeutic index of the anticancer drug by increasing pharmaceutical bioavailability and targeting. However, the formation of protein corona largely affects biological fate, pharmacokinetics, and cellular interactions of nanomaterials. Next, we investigated the physical changes occurring in GE11-LPs after protein corona formation. As shown in Figure 1(b), GE11-LPs were smaller to homogeneous size when dispersed in PBS, while a relatively larger particle size with heterogeneous size distribution was observed after incubation with culture medium, which could be attributed to protein corona formation. To further demonstrate the presence of protein corona, the adsorbed proteins were separated from GE11-LPs and identified by SDS PAGE. The results showed that the protein composition from the culture medium was reserved in GE11-LPs, but no protein signal was detected in naked GE11-LPs (Figure 1(g)), further indicating the retention of protein corona on the surface of GE11-LPs.

**Figure 2:**
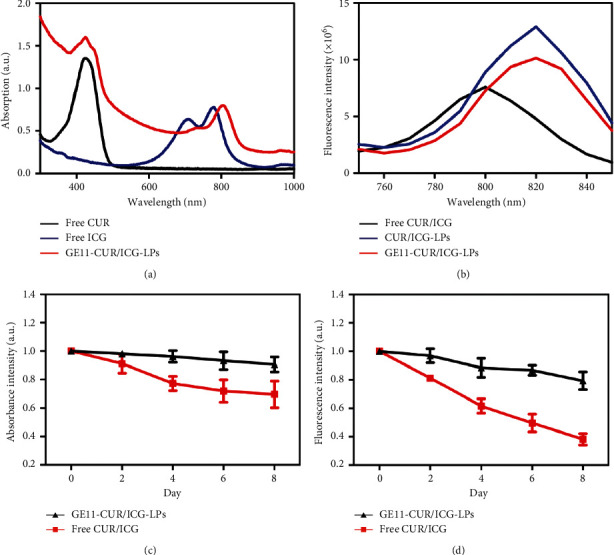
(a) Absorption spectrum of free ICG, free CUR, and GE11-CUR/ICG-LPs. (b) Fluorescence spectrum of free CUR/ICG, CUR/ICG-LPs, and GE11-CUR/ICG-LPs with the excitation wavelength of 700 nm. (c) The absorbance and (d) fluorescence changes of free CUR/ICG and GE11-CUR/ICG-LPs.

The UV-Vis absorption spectrum of GE11-CUR/ICG-LPs (Figure 2(a)) exhibits two characteristic absorption peaks. The absorption peak at 416 nm is a characteristic absorption for CUR and the second absorption peak at ∼770 nm is a characteristic absorption for ICG, indicating the successful coencapsulation of ICG and CUR into GE11-LPs. Compared with free ICG, the absorption or emission peaks of ICG in CUR/ICG-LPs and GE11-CUR/ICG-LPs were red-shifted by ∼26 and ∼20 nm, respectively (Figure 2(b)), possibly due to the changes in molecular conformation caused by the interactions between ICG and CUR. The red-shifted absorption peak close to 808 nm would be beneficial for GE11-CUR/ICG-LPs applied in NIR-mediated phototherapy. Free ICG in an aqueous solution tends to aggregate to form dimers and oligomers, resulting in fluorescence self-quenching [36]. Interestingly, there were no significant fluorescence or absorbance changes of the GE11-CUR/ICG-LPs under 37°C over 8 days, while free CUR/ICG almost completely degraded to show only 38.2% of its initial fluorescence intensity and 69.6% of its initial absorbance intensity after 8 days (Figures 2(c) and 2(d)). These results indicated the excellent optical stability of GE11-CUR/ICG-LPs, which was helpful to stabilize ICG by isolating it from aggregates, therefore reducing ICG quenching.

### 3.2. Photothermal Properties and Light-Triggered Drug Release of GE11-CUR/ICG-LPs

**Figure 3:**
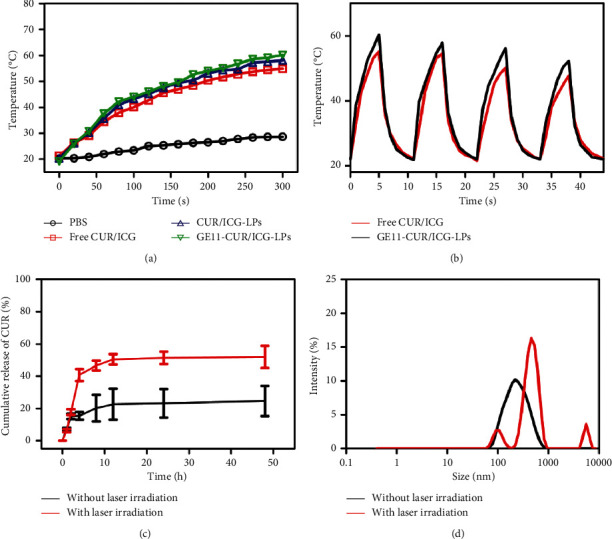
(a) Temperature elevation curves of PBS, free CUR/ICG, CUR/ICG-LPs, and GE11-CUR/ICG-LPs under laser irradiation (1 W·cm^−2^, 5 min). (b) The temperature change of free CUR/ICG and GE11-CUR/ICG-LPs over four cycles of repeated laser irradiation (1 W·cm^−2^, 5 min/cycles). (c) In vitro release profiles of CUR from GE11-CUR/ICG-LPs with or without 808 nm NIR irradiation at pH 7.4. (d) DLS profiles of GE11-CUR/ICG-LPs before and after NIR irradiation at 808 nm for 5 min.

Due to the strong absorption of ICG in the NIR region, the photothermal potency of free CUR/ICG, CUR/ICG-LPs, and GE11-CUR/ICG-LPs was evaluated by monitoring the temperature changes using an infrared thermal imaging camera. After laser irradiation at 808 nm (1.0 W·cm^−2^) for 5 min, the temperature of PBS slightly increased to 28.7°C, while free CUR/ICG, CUR/ICG-LPs, and GE11-CUR/ICG-LPs showed a quick increase trend to a maximal temperature of 54.9°C, 58.1°C, and 60.2°C, respectively (Figure 3(a)). It was also easily found that CUR/ICG-LPs and GE11-CUR/ICG-LPs formulation showed slightly higher photothermal efficiency than free CUR/ICG under laser irradiation, which might be mainly ascribed to the reduced heat dissipation protected by lipid bilayers membrane and the enhanced laser energy absorption caused by red-shifted absorbance peak. Furthermore, to explore the photothermal stability of free CUR/ICG and GE11-CUR/ICG-LPs, four cycles of laser on/off with 808 nm laser irradiation (1.0 W·cm^−2^) were performed. As shown in Figure 3(b), the temperature elevation of free CUR/ICG was gradually decreased with repeated irradiation cycles. However, GE11-CUR/ICG-LPs showed consistent temperature changes over four cycles, indicating much better photostability of GE11-CUR/ICG-LPs beyond the free CUR/ICG. Together, such excellent photothermal efficiency and photostability enable great potentials of GE11-CUR/ICG-LPs for further photothermal therapy.

In addition, we further investigated the release behavior of CUR from GE11-CUR/ICG-LPs before and after NIR irradiation. As shown in Figure 3(c), the GE11-CUR/ICG-LPs without laser irradiation was released slowly and the total release of CUR only reached 21.51% in 48 h, indicating that GE11-LPs could efficiently hinder the leakage of encapsulated CUR. However, when exposed to NIR laser irradiation, the release of CUR from GE11-LPs was accelerated to 40.8% in the first 4 h and was finally enhanced to 52.0% in 48 h, which suggested that the drug release of GE11-CUR/ICG-LPs could be triggered by laser irradiation. Based on these results, we, therefore, supposed that the GE11-CUR/ICG-LPs system might be destroyed by the hyperthermia produced by ICG under NIR irradiation, which led to the burst release of entrapped CUR. The uneven size distribution of GE11-CUR/ICG-LPs after laser irradiation further confirmed the assumption that the thermal destruction of GE11-CUR/ICG-LPs induced by ICG hyperthermia (Figure 3(d)). These results collectively suggested that the CUR released from GE11-CUR/ICG-LPs could be controllable by NIR laser irradiation, which highlights GE11-CUR/ICG-LPs as a kind of NIR-sensitive drug release nanosystem.

### 3.3. Cellular Uptake and Light-Triggered Intracellular Drug Release of GE11-CUR/ICG-LPs

**Figure 4:**
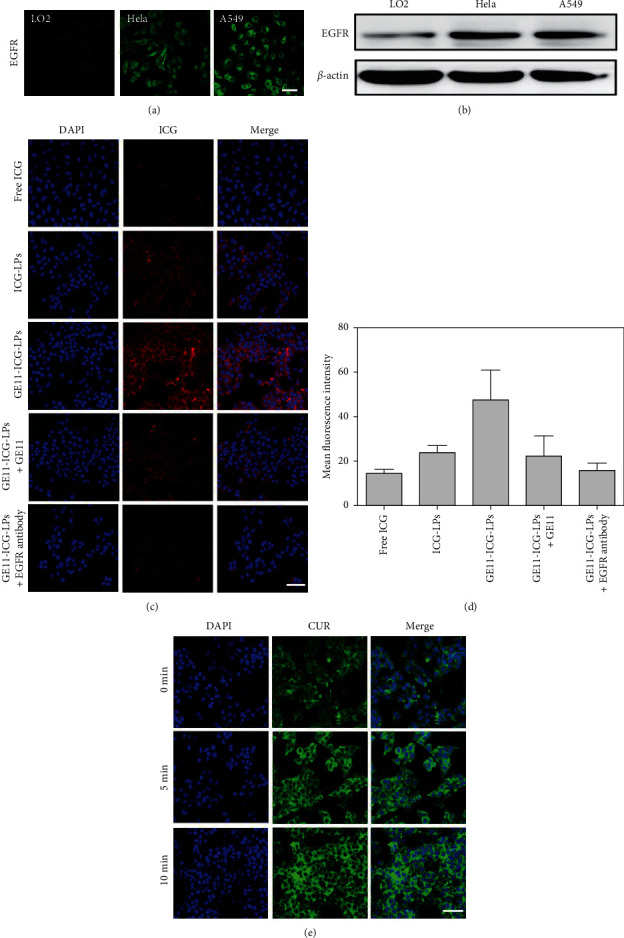
(a) Immunocytochemical staining and (b) protein level of EGFR expression in LO2 cells, HeLa cells, and A549 cells. Scale bar = 50 *µ*m. (c) FL images of A549 cells after 4 h of incubation with free ICG, ICG-LPs, GE11-ICG-LPs, and GE11-ICG-LPs with free GE11 peptide or anti-EGFR antibody blocking. DAPI (blue) counterstains cell nuclei. Scale bar = 50 *µ*m. (d) The semiquantitative analysis of fluorescence intensity in (c) was determined by ‘Image J' software. Data are expressed as mean ± SD (*n* = 3). (e) FL images of A549 cells incubated with GE11-CUR/ICG-LPs after laser irradiation (1 W·cm^−2^) for different times. Scale bar = 50 *µ*m.

Epidermal growth factor receptor (EGFR) is known to be overexpressed in nonsmall cell lung cancer and therefore widely served as an important therapeutic target, which has attracted enormous interest in cancer treatment [37, 38]. Our and other groups' works have introduced GE11 peptide as a cancer targeting ligand specificity against EGFR proteins [19, 39, 40]. By conjugating GE11 peptide onto the surface of LPs, the obtained LPs would actively target EGFR-high expressing tumor cells and synchronously evade EGFR-low expressing normal cells for enhanced anticancer effects and reduced off-target effects. Firstly, we investigated EGFR expression in HeLa cells and A549 cells with control LO2 normal cells. The results showed that EGFR was expressed most strongly in A549 cells and least in LO2 cells, as shown by the immunofluorescence staining (Figure 4(a)) and western blot analysis (Figure 4(b)). Therefore, EGFR highly expressed A549 cells were selected to further examine the specific targeting effect of GE11-LPs mediated by GE11 peptides. As shown in Figures 4(c) and 4(d), A549 cells treated with GE11-ICG-LPs showed stronger ﬂuorescence than those treated with free ICG or ICG-LPs, which indicated that GE11-ICG-LPs were more easily guided and to be captured by EGFR overexpressed A549 cells due to the assistance of GE11 peptide targeting effects.

To further verify the contribution of GE11 peptides in the uptake of GE11-ICG-LPs, the competing assay was performed in A549 cells. The cells were pretreated with an excess amount of GE11 peptides or anti-EGFR antibody to block specific binding of the GE11-ICG-LPs. The uptake of GE11-ICG-LPs was markedly inhibited to a degree similar to that achieved by ICG-LPs. More notably, anti-EGFR antibody blocking also caused the reduced targeting effects of GE11-ICG-LPs, which further indicated that the targeted uptake process of GE11-ICG-LPs was mainly mediated by EGFR-targeted GE11 peptides. Together, these results confirmed that GE11 modified LPs could specifically and actively target to EGFR-positive A549 tumor cells. High transport efficiency of GE11-LPs would contribute to the increased drug contents at the tumor site, thereby improving antitumor effects and reducing their potential side effects.

We further assessed light-triggered release behavior of GE11-CUR/ICG-LPs inside the tumor cells. The cells incubated with GE11-CUR/ICG-LPs were exposed to 808 nm laser irradiation (1 W·cm^−2^) for different times; after that, the fluorescence signal of CUR releasing from the GE11-CUR/ICG-LPs was visualized by confocal microscopy. As exhibited in Figure 4(e), the fluorescence intensities of CUR were relatively low before irradiation while gradually increased with the prolonged irradiation time. These results indicated that similar to the NIR irradiation triggered CUR release from GE11-CUR/ICG-LPs in PBS, laser irradiation also observably increased the release rate of CUR from GE11-CUR/ICG-LPs inside cancer cells. These results further demonstrated that heat induced by ICG decomposed the intracellular GE11-LPs systems for fast release of CUR during laser irradiation, which, therefore, potentially combines the photothermal therapy and chemotherapy for enhanced anticancer treatment.

### 3.4. In Vitro Cytotoxicity and Phototoxicity of GE11-CUR/ICG-LPs

**Figure 5:**
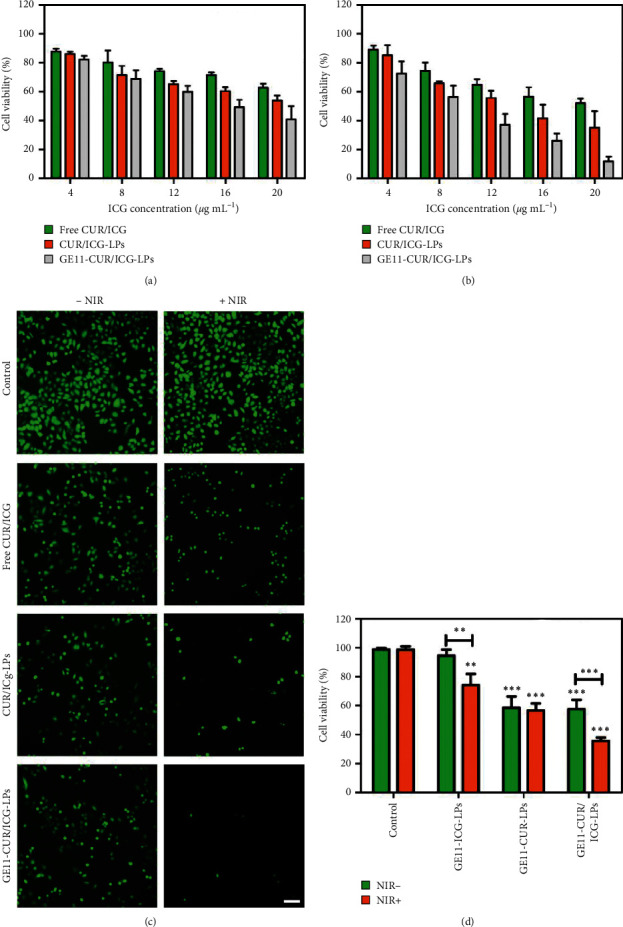
(a), (b) Cytotoxicity and phototoxicity of free CUR/ICG, CUR/ICG-LPs and GE11-CUR/ICG-LPs under the NIR laser irradiation (808 nm) of 1 W·cm^−2^ for 5 min at different concentrations on A549 cells after 24 h incubation. (c) Fluorescence images of A549 cells stained with calcein-AM (green) after different treatments. Scale bar = 100 *μ*m. (d) Synergistic effect of photo- and chemotherapy based on GE11-CUR/ICG-LPs. Cytotoxicity of GE11-ICG-LPs, GE11-CUR-LPs, and GE11-CUR/ICG-LPs with or without laser exposure was performed by MTT assays. Data are expressed as mean ± SD (*n* = 3). ^*∗*^*P* < 0.05, ^*∗∗*^*P* < 0.01 and ^*∗∗∗*^*P* < 0.001.

The therapeutic potentials of GE11-CUR/ICG-LPs against A549 cells were further verified by MTT assay and calcein-AM staining assays. Without NIR irradiation, the enhanced cell viability inhibition by GE11-CUR/ICG-LPs than free CUR/ICG or CUR/ICG-LPs might be attributed to the improved intracellular accumulation of CUR, which thereby resulted in more efficient cytotoxicity (Figure 5(a)). After irradiation, the growth tendency of the cells was similar to that of the cells treated without irradiation. The viability of cells treated with free CUR/ICG or CUR/ICG-LPs treatment (25 *μ*g·mL^−1^ for CUR and 20 *μ*g·mL^−1^ for ICG) decreased to 52.0% or 35.2%, respectively, while GE11-CUR/ICG-LPs significantly reduced the cell viability to 12% (Figure 5(b)), indicating the stronger cytotoxicity and phototoxicity of GE11-CUR/ICG-LPs against cancer cells.

It was notable that GE11-CUR/ICG-LPs showed higher cytotoxicity than CUR/ICG-LPs at almost all evaluated concentrations, which might be attributed to its stronger cellular internalization in A549 cells by the GE11 targeting effects against EGFR. To further confirm the therapeutic outcomes, calcein-AM staining was used to visualize living cells with different treatments. Consistent with the MTT results, it further proved that cells treated with GE11-CUR/ICG-LPs + NIR induced the strongest cancer cell killing effects while other groups exhibited relatively lower cytotoxicity (Figure 5(c)).

To examine the synergistic effects of photochemotherapy combined treatments, we explored the anticancer effects of GE11-ICG-LPs, GE11-CUR-LPs, and GE11-CUR/ICG-LPs under light exposure. As expected, the combination of GE11-CUR/ICG-LPs and laser irradiation exerts stronger cytotoxicity than either GE11-ICG-LPs or GE11-CUR-LPs treatment alone (Figure 5(d)). We, therefore, infer that NIR light-controlled GE11-CUR/ICG-LPs could generate sufficient heat energy for photothermal therapy, which then accelerated the release of CUR for combined chemotherapy, achieving inspiring synergetic inhibition of cancer cells. The heat production and CUR release were strictly controlled by NIR light, greatly minimizing off-target drug exposure. In addition, there was no significant difference in GE11-CUR-LPs treated cells before or after irradiation, indicating the important roles of ICG mediated phototherapy. Taken together, these light-controlled EGFR-targeted nanocarriers would be a promising strategy to combine chemophotothermal therapy for synergetic cancer killing with low side effects.

### 3.5. Cancer Cell Apoptosis Induced by GE11-CUR/ICG-LPs

**Figure 6:**
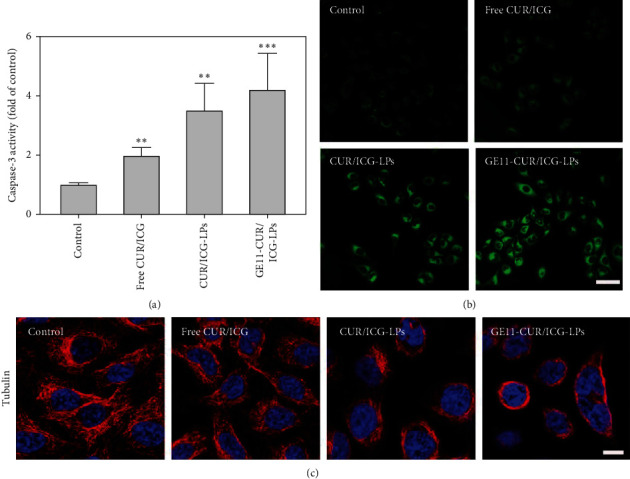
(a) Effects of IC-GLPs on the caspase-3 activity in A549 cells, data are expressed as mean ± SD (*n* = 3). ^*∗*^*P* < 0.05, ^*∗∗*^*P* < 0.01 and ^*∗∗∗*^*P* < 0.001. (b) Intracellular ROS detection by the DCFH-DA fluorescence staining. Scale bar = 50 *μ*m. (c) Cytoskeletal microtubulin (red) expression in A549 cells after different treatments with laser irradiation. DAPI (blue) counterstains cell nuclei. Scale bar = 10 *μ*m.

Apoptosis has been considered as one of the most important mechanisms for cell death [41]. Caspases family plays a central role in the apoptotic process, which is widely used as indicators to monitor apoptosis [42]. To verify whether apoptosis contributed to the A549 cell proliferation inhibition induced by GE11-CUR/ICG-LPs, the activity of caspase-3 was detected after NIR irradiation. As seen in Figure 6(a), the caspase-3 activity of A549 cells increased from 0.98 for control cells to 1.94, 3.48, and 4.18 for NIR irradiated free CUR/ICG, CUR/ICG-LPs, and GE11-CUR/ICG-LPs treated cells, respectively, which revealed that GE11-CUR/ICG-LPs + NIR could significantly induce apoptosis in a caspase-dependent manner. Thus, these results, consistent with the MTT results, further indicated that coencapsulation of ICG and CUR into GE11 coated LPs could achieve significantly stronger cell apoptosis due to the targeted delivery and synergetic effects of CUR and ICG in GE11-CUR/ICG-LPs system.

Intracellular ROS levels are considered to be a reliable indicator of oxidative stress, which is essential for apoptosis induction [43]. Therefore, the ROS levels of A549 cells treated with respective formulations upon laser irradiation were further evaluated. As shown in Figure 6(b), no remarkable increase in ROS generation was observed in control cells, further indicating that photothermal irradiation would not cause obvious damage in untreated cells. Moreover, free CUR/ICG treatment slightly increased intracellular ROS levels, while there was a remarkable increase of ROS generation in cells treated with GE11-CUR/ICG-LPs, which suggested that the ROS overproduction was closely related to GE11-CUR/ICG-LPs + NIR induced apoptosis.

Cell apoptosis is always accompanied by cell morphological changes. Hence, we sought to visualize the changes in microtubule skeleton by immunofluorescence analysis. As shown in Figure 6(c), the untreated cells showed spindly contour and integrated microtubule fibers, however, free CUR/ICG and CUR/ICG-LPs treatment upon NIR irradiation induced similar damage of microtubule structure in cells, where thread-like fibers decreased gradually in the cytoplasm. Furthermore, the morphology of cells treated with GE11-CUR/ICG-LPs became shrink and the microtubule fragments were greatly disappeared, further suggesting the excellent cytotoxicity induced by GE11-CUR/ICG-LPs and NIR irradiation.

**Figure 7:**
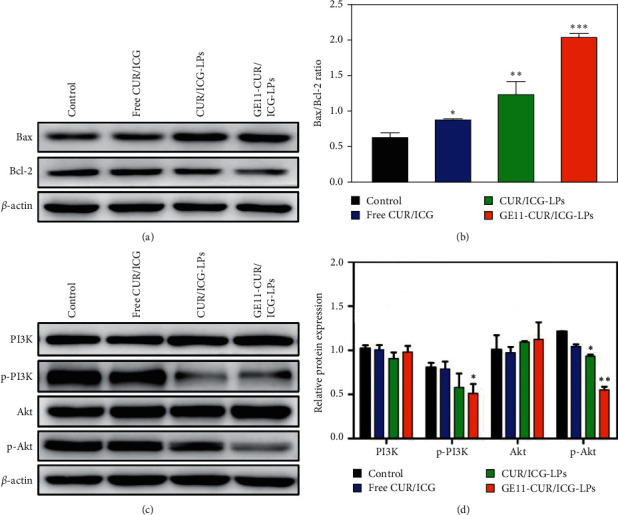
(a) Representative western blot and (b) quantification of Bcl-2 and Bax expression in A549 cells following treatment with PBS, free CUR/ICG, CUR/ICG-LPs, and GE11-CUR/ICG-LPs under 808 nm laser irradiation for 5 min. Data are expressed as mean ± SD (*n* = 3). ^*∗*^*P* < 0.05 and ^*∗∗*^*P* < 0.01. (c) Representative western blot and (d) quantification of PI3K, p-PI3K, Akt, and p-Akt expression in A549 cells following different treatments with laser irradiation. Data are expressed as mean ± SD (*n* = 3). ^*∗*^*P* < 0.05, ^*∗∗*^*P* < 0.01 and ^*∗∗∗*^*P* < 0.001.

To further explore the underlying mechanism of apoptosis, Bcl-2 family proteins were detected by western blot analysis. Bcl-2 is widely identified as a crucial antiapoptotic molecular signature, while Bax is specifically considered as an important proapoptotic molecular signature, which plays an essential role in regulating and controlling the apoptotic signaling pathway [44, 45]. As shown in Figure 7(a), the expression of Bax protein increased significantly after treatment with CUR/ICG-LPs and GE11-CUR/ICG-LPs formulation with NIR irradiation beyond free CUR/ICG with NIR irradiation, while an opposite tendency was observed in the expression of Bcl-2. GE11-CUR/ICG-LPs + NIR irradiation group exhibited a ∼1.6-fold increase in Bax/Bcl-2 ratio compared with the CUR/ICG-LPs + NIR irradiation group, and a ∼2.3-fold increase compared with the free ICG/NIR irradiation group (Figure 7(b)). The superior anticancer effects of GE11-CUR/ICG-LPs could be attributed to high intracellular drug concentrations and excellent photothermal efficiency.

EGFR-downstream PI3K/AKT signaling pathway is one of the most significant pathways potentially influencing plenty of cellular processes including growth, proliferation, metabolism, and autophagy [46]. The regulation of the PI3K/AKT signaling pathway is intimately associated with the protection of cells against apoptosis [47]. Therefore, we next determined whether GE11-CUR/ICG-LPs + NIR irradiation could impact PI3K/AKT signaling in A549 cells. As displayed in Figures 7(c) and 7(d), although there were no obvious changes in PI3K and AKT expression levels, the phosphorylation of PI3K and AKT levels downregulated obviously after treatment with free CUR/ICG, CUR/ICG-LPs, and GE11-CUR/ICG-LPs followed by NIR irradiation. GE11-CUR/ICG-LPs + NIR treatment showed the strongest inhibition in the phosphorylation level of PI3K and AKT in comparison with other treatments. Thus, these results further verified that GE11-CUR/ICG-LPs could combine chemophotothermal treatments to induce enhanced cancer cell apoptosis through the activation of apoptotic signaling pathways and the inhibition of EGFR-mediated PI3K/AKT pathway.

## 4. Conclusion

In summary, NIR-sensitive and EGFR-targeted GE11-CUR/ICG-LPs were developed in this work through coencapsulation of ICG and CUR into GE11 peptides conjugated liposomes for a synergistic combination of chemophototherapy. The proposed GE11-CUR/ICG-LPs could not only actively target EGFR overexpressed cancer cells but also enable selective drug release controlled by NIR light, to significantly enhance the drug concentration at tumor sites, which could significantly reduce the off-target effects of CUR. Upon 808 nm NIR laser irradiation, the hyperthermia generated by ICG could ablate the tumor robustly; and, on the other hand, could also trigger the release of CUR from GE11-CUR/ICG-LPs to eradicate residual cancer cells, thus achieving synergetic antitumor effects. Our results also revealed that GE11-CUR/ICG-LPs with NIR irradiation could induce cancer cell apoptosis by promoting ROS generation and cytoskeleton disruption through the activation of apoptotic signaling pathways and the inhibition of EGFR-mediated PI3K/AKT pathway. Taken together, this NIR controlled and cancer targeted design of GE11-CUR/ICG-LPs nanosystem presents a novel strategy for chemohyperthermic anticancer treatment.

## Data Availability

The data used to support the findings of this study are available from the corresponding author upon request.
